# Analysis of the association between atrial fibrillation with in-hospital mortality in people admitted for community-acquired pneumonia through an observational, nation-wide, sex-stratified study

**DOI:** 10.1038/s41598-022-18810-8

**Published:** 2022-08-24

**Authors:** José M. de-Miguel-Yanes, Rodrigo Jiménez-García, Valentín Hernández-Barrera, Javier de-Miguel-Díez, Manuel Méndez-Bailón, Ana López-de-Andrés

**Affiliations:** 1 Internal Medicine Department, Instituto de Investigación Sanitaria Gregorio Marañón (IiSGM), Hospital General Universitario Gregorio Marañón, Universidad Complutense de Madrid, 46, Doctor Esquerdo, 28007 Madrid, Spain; 2 Department of Public Health and Maternal and Child Health, Faculty of Medicine, Universidad Complutense de Madrid, Madrid, Spain; 3 Preventive Medicine and Public Health Teaching and Research Unit, Department of Medical Specialties and Public Health, Universidad Rey Juan Carlos, Alcorcón, Madrid, Spain; 4 Respiratory Care Department, Instituto de Investigación Sanitaria Gregorio Marañón (IiSGM), Hospital General Universitario Gregorio Marañón, Universidad Complutense de Madrid, Madrid, Spain; 5 Internal Medicine Department, Hospital Universitario Clínico San Carlos, Universidad Complutense de Madrid, Madrid, Spain

**Keywords:** Medical research, Epidemiology

## Abstract

We aimed to analyze the influence of atrial fibrillation (AF) prior to hospital admission (“prevalent”) and AF diagnosed during hospital admission (“incident”) on in-hospital mortality (IHM) in women and men admitted for community-acquired pneumonia (CAP) in Spain (2016–2019). We used the Spanish Register of Specialized Care‐Basic Minimum Database. We analyzed 519,750 cases of CAP in people ≥ 18 years (213,631 women (41.1%)), out of which people with prevalent AF represented 23.75% (N = 123,440), whereas people with incident AF constituted 0.60% (N = 3154). Versus no AF, crude IHM was significantly higher for prevalent AF (15.24% vs. 11.40%, *p* < 0.001) and for incident AF (23.84% vs. 12.24%, *p* < 0.001). After propensity score marching, IHM in women and men with prevalent AF neared IHM in women and men with no AF (15.72% vs. 15.52%, *p* = 0.425; and 14.90% vs. 14.99%, *p* = 0.631, respectively), but IHM in women and men with incident AF was higher than IHM in women and men with no AF (24.37% vs. 13.36%, *p* < 0.001; and 23.94% vs. 14.04%, *p* < 0.001, respectively). Male sex was associated with a higher IHM in people with prevalent AF (OR 1.06; 95% CI 1.02–1–10), but not in people with incident AF (OR 0.93; 95% CI 0.77–1–13). AF diagnosed during hospital admission was associated with a higher IHM, irrespectively of sex.

## Introduction

Community-acquired pneumonia (CAP) continues to be a common indication for hospital admission, especially among adults with underlying clinical risk conditions, and because of the increasing number of comorbidities in an ageing population^1,2^. Different severity indexes have proven their clinical usefulness, as they allow a prompt risk stratification at the emergency room^3^. Regrettably, these indexes have been reported to have limitations^4^. Indeed, clinical practice is dynamic and has consequently changed since the publication of these scores. For instance, non-invasive ventilation is now more routinely applied out of the intensive care environment^5^.

The onset of arrhythmias during hospital admission for CAP is a well-known complication and has been reported to be around 4–9%^6,7^. Recent research work has raised that number to an astonishing 10% in the case of *Streptococcus pneumoniae* infection and has additionally shown an association between new-onset atrial fibrillation and in-hospital mortality (IHM)^8^. Older research had formerly found an association between incident atrial fibrillation and IHM, as well^9,10^. In the paper by Ruiz et al., an association was also found between atrial fibrillation present at hospital admission that persisted during the hospital stay and IHM^8^. Nonetheless, previous reports had failed to show an association between chronic atrial fibrillation and IHM in people admitted for CAP^7^.

Furthermore, sex may influence the outcomes of CAP^11^. Although cultural, behavioural, and socio-economic differences may be important determinants to explain the effect exerted by gender on the clinical management and outcomes of pneumonia^12^, a sex gap due to biological differences cannot be ruled out^13^. The interplay between sex, pneumonia and prevalent or incident atrial fibrillation is therefore complex, and few studies with large population sizes have focused on atrial fibrillation as a specific condition in separate analyses for men and women admitted for pneumonia.

Here we aimed to compare the clinical characteristics and in-hospital outcomes for women and men with CAP needing admission to the hospital during the extended period 2016–2019 in Spain according to the presence of atrial fibrillation prior to hospital admission, and new onset atrial fibrillation during hospital admission. We used propensity score matching (PSM) with the purpose of attenuating baseline differences for the comparisons. We finally sought the variables associated with IHM for patients admitted for CAP with atrial fibrillation prior to hospital admission or new onset atrial fibrillation during hospital admission according to sex.

## Results

### Clinical characteristics and in-hospital outcomes for the overall population according to atrial fibrillation prior to hospital admission status

A total of 519,750 cases of CAP in people ≥ 18 years were admitted to Spanish hospitals during the study period (Table 1), out of which 213,631 cases corresponded to women (41.1%). Overall, people coded for atrial fibrillation prior to hospital admission represented 23.75% (N = 123,440) of the population. Women constituted 41.71% of the population with prior history of atrial fibrillation, whereas the proportion of women in the population with no prior history of atrial fibrillation was 40.91%.

**Table 1:** Clinical characteristics, and in-hospital outcomes of patients hospitalized with community-acquired pneumonia in Spain from 2016 to 2019 according to atrial fibrillation prior to hospital admission, and to atrial fibrillation diagnosed during hospital admission.

		Atrial fibrillation prior to hospital admission			Atrial fibrillation diagnosed during hospital admission		
		Yes	No	*p* value	Yes	No	*p* value
Sex, N(%)	Male	71,959 (58.29)	234,160 (59.09)	< 0.001	1954 (61.95)	304,165 (58.88)	< 0.001
	Female	51,481 (41.71)	162,150 (40.91)		1200 (38.05)	212,431 (41.12)	
Age, mean (SD)		82.14 (8.95)	71.99 (16.46)	< 0.001	76.55 (11.69)	74.39 (15.65)	< 0.001
Age category, N(%)	18–54	1035 (0.84)	62,190 (15.69)	< 0.001	132 (4.19)	63,093 (12.21)	< 0.001
	55–69	10,376 (8.41)	85,757 (21.64)		683 (21.66)	95,450 (18.48)	
	70–84	55,697 (45.12)	146,487 (36.96)		1441 (4.69)	200,743 (38.86)	
	≥ 85	56,332 (45.64)	101,876 (25.71)		898 (28.47)	157,310 (30.45)	
Charlson comorbidity index, mean (SD)		2.45 (1.84)	2.02 (2.04)	< 0.001	2.55 (2.04)	2.12 (2.00)	< 0.001
Prior myocardial infarction, N(%)	Present	7922 (6.42)	17,350 (4.38)	< 0.001	263 (8.34)	25,009 (4.84)	< 0.001
Prior congestive heart failure, N(%)	Present	55,522 (44.98)	61,496 (15.52)	< 0.001	1156 (36.65)	115,862 (22.43)	< 0.001
Prior peripheral vascular disease, N(%)	Present	8503 (6.89)	19,057 (4.81)	< 0.001	230 (7.29)	27,330 (5.29)	< 0.001
Prior cerebrovascular disease, N(%)	Present	10,644 (8.62)	22,178 (5.6)	< 0.001	228 (7.23)	32,594 (6.31)	0.034
Dementia, N(%)	Present	12,014 (9.73)	34,649 (8.74)	< 0.001	186 (5.90)	46,477 (9.00)	< 0.001
Prior Chronic obstructive pulmonary disease, N(%)	Present	42,024 (34.04)	121,854 (30.75)	< 0.001	1049 (33.26)	162,829 (31.52)	0.036
Type 2 diabetes mellitus, N(%)	Present	40,769 (33.03)	99,434 (25.09)	< 0.001	929 (29.45)	139,274 (26.96)	0.002
Prior rheumatoid disease, N(%)	Present	3445 (2.79)	10,116 (2.55)	< 0.001	88 (2.79)	13,473 (2.61)	0.522
Prior peptic ulcer, N(%)	Present	696 (0.56)	2252 (0.57)	0.857	31 (0.98)	2917 (0.56)	0.002
Prior liver disease, N(%)	Present	5877 (4.76)	25,293 (6.38)	< 0.001	225 (7.13)	30,945 (5.99)	0.007
Prior hemiplegia or paraplegia, N(%)	Present	876 (0.71)	3399 (0.86)	< 0.001	29 (0.92)	4246 (0.82)	0.545
Prior renal disease, N(%)	Present	33,679 (27.28)	62,232 (15.7)	< 0.001	711 (22.54)	95,200 (18.43)	< 0.001
Cancer, N(%)	Present	12,200 (9.88)	56,964 (14.37)	< 0.001	534 (16.93)	68,630 (13.29)	< 0.001
Acquired Immunodeficiency syndrome, N(%)	Present	123 (0.10)	5033 (1.27)	< 0.001	12 (0.38)	5144 (1.00)	0.001
Undergone any surgery, N(%)	Present	2905 (2.35)	11,372 (2.87)	< 0.001	370 (11.73)	13,907 (2.69)	< 0.001
Bronchial fibroscopy, N(%)	Yes	926 (0.75)	4996 (1.26)	< 0.001	73 (2.31)	5849 (1.13)	< 0.001
Chest computed tomography, N(%)	Yes	5859 (4.75)	25,246 (6.37)	< 0.001	294 (9.32)	30,811 (5.96)	< 0.001
Dialysis, N(%)	Yes	1395 (1.13)	4204 (1.06)	0.039	184 (5.83)	5415 (1.05)	< 0.001
Oxygen prior to admission, N(%)	Present	10,173 (8.24)	23,012 (5.81)	< 0.001	183 (5.80)	33,002 (6.39)	0.179
Non-invasive lung ventilation, N(%)	Yes	3940 (3.19)	10,724 (2.71)	< 0.001	372 (11.79)	14,292 (2.77)	< 0.001
Invasive lung ventilation, N(%)	Yes	2576 (2.09)	10,374 (2.62)	< 0.001	534 (16.93)	12,416 (2.40)	< 0.001
Days of hospital stay, median (IQR)		8 (7)	7 (7)	< 0.001	12 (12)	7 (7)	< 0.001
In hospital mortality, N(%)	Yes	18,814 (15.24)	45,195 (11.40)	< 0.001	752 (23.84)	63,257 (12.24)	< 0.001

People with atrial fibrillation prior to hospital admission were older than people without atrial fibrillation (82.14 ± 8.95 vs. 71.99 ± 16.46 years; *p* < 0.001) and with more comorbidities (*p* < 0.001). They had more commonly suffered from cardiovascular conditions, dementia, chronic obstructive pulmonary disease, type 2 diabetes mellitus, renal disease, and rheumatoid disease, and were reported to have a higher percentage of oxygen use at home as well (all *p* values < 0.001) (Table 1). People with atrial fibrillation prior to hospital admission less often underwent bronchial fibroscopy, chest computed tomography and invasive lung ventilation during the hospitalization period than people without atrial fibrillation (all p values < 0.001) but had a higher probability of receiving non-invasive ventilation (*p* < 0.001) (Table 1). Length of hospital stay was similar between both groups (8 ± 7 vs. 7 ± 7 days), yet crude IHM was significantly higher in people with atrial fibrillation prior to hospital admission (15.24% vs. 11.40%; *p* < 0.001).

### Clinical characteristics and in-hospital outcomes for the overall population according to atrial fibrillation diagnosed during hospital admission status

People with new onset of atrial fibrillation during hospital admission represented 0.60% (N = 3154) of the total population. The proportion of women with atrial fibrillation diagnosed during hospital admission was 38.05%, vs. 41.12% in the population without this condition.

People with atrial fibrillation diagnosed during hospital admission were older than people without atrial fibrillation (76.55 ± 11.69 vs. 74.39 ± 15.65 years; *p* < 0.001) and had more comorbidities (*p* < 0.001). They had more commonly suffered from cardiovascular conditions (all p values < 0.001, except for cerebrovascular disease (*p* = 0.034)), chronic obstructive pulmonary disease (*p* = 0.036), type 2 diabetes mellitus (*p* = 0.002), liver disease (*p* = 0.007), renal disease (*p* = 0.001), and cancer (*p* = 0.001) (Table 1). People with atrial fibrillation diagnosed during hospital admission more often underwent bronchial fibroscopy, chest computed tomography, dialysis, and both non-invasive and invasive lung ventilation than people without atrial fibrillation (all p values < 0.001) (Table 1). Both length of hospital stay (12 ± 12 vs. 7 ± 7 days), and crude IHM (23.84% vs. 12.24%; *p* < 0.001) were higher in people with atrial fibrillation diagnosed during hospital admission than in people without atrial fibrillation.

### Clinical characteristics and in-hospital outcomes for women and men by atrial fibrillation prior to hospital admission status after propensity score matching

After PSM, women with atrial fibrillation prior to hospital admission had more often suffered from heart failure (*p* = 0.003), peripheral vascular disease (*p* = 0.028), cerebrovascular disease (*p* < 0.001), and more frequently used oxygen at home (*p* = 0.001) (Table 2). Despite they more commonly received both non-invasive and invasive lung ventilation (both *p* < 0.001), their IHM did not differ from IHM in women with no atrial fibrillation (15.72% vs. 15.52%; *p* = 0.425).

**Table 2:** Clinical characteristics, and in-hospital outcomes of women hospitalized with community-acquired pneumonia in Spain from 2016 to 2019 according to atrial fibrillation prior to hospital admission, before and after propensity score matching.

		Before propensity score matching			After propensity score matching		
		Atrial fibrillation prior to hospital admission			Atrial fibrillation prior to hospital admission		
		Yes	No	*p* value	Yes	No	*p* value
Age, mean(SD)		84.08 (8.30)	73.2 (17.25)	< 0.001	84.08 (8.30)	84.32 (8.50)	0.623
Age category, N(%)	18–54	235 (0.46)	25,297 (15.60)	< 0.001	235 (0.46)	235 (0.46)	0.339
	55–69	2866 (5.57)	31,599 (19.49)		2860 (5.56)	2841 (5.52)	
	70–84	19,977 (38.8)	53,440 (32.96)		19,955 (38.80)	19,685 (38.27)	
	≥ 85	28,403 (55.17)	51,814 (31.95)		28,384 (55.19)	28,673 (55.75)	
Charlson comorbidity index, mean (SD)		2.17 (1.64)	1,68 (1.81)	< 0.001	2.17 (1.64)	2.19 (1.71)	0.432
Prior myocardial infarction, N (%)	Present	2022 (3.93)	4017 (2.48)	< 0.001	2019 (3.93)	2084 (4.05)	0.300
Prior congestive heart failure, N (%)	Present	26,053 (50.61)	28,388 (17.51)	< 0.001	26,025 (50.60)	25,546 (49.67)	0.003
Prior peripheral vascular disease, N (%)	Present	1656 (3.22)	3504 (2.16)	< 0.001	1653 (3.21)	1531 (2.98)	0.028
Prior cerebrovascular disease, N (%)	Present	4377 (8.50)	7682 (4.74)	< 0.001	4370 (8.50)	3258 (6.33)	< 0.001
Dementia, N (%)	Present	6422 (12.47)	17,766 (10.96)	< 0.001	6416 (12.47)	6800 (13.22)	< 0.001
Prior Chronic obstructive pulmonary disease, N (%)	Present	11,016 (21.40)	36,851 (22.73)	< 0.001	11,006 (21.40)	10,896 (21.18)	0.402
Type 2 diabetes mellitus, N (%)	Present	16,705 (32.45)	36,736 (22.66)	< 0.001	16,690 (32.45)	16,634 (32.34)	0.709
Prior rheumatoid disease, N (%)	Present	1830 (3.55)	6139 (3.79)	0.016	1827 (3.55)	1955 (3.80)	0.034
Prior peptic ulcer, N (%)	Present	212 (0.41)	647 (0.40)	0.690	212 (0.41)	227 (0.44)	0.473
Prior liver disease, N (%)	Present	1777 (3.45)	7369 (4.54)	< 0.001	1773 (3.45)	1732 (3.37)	0.481
Prior hemiplegia or paraplegia, N (%)	Present	367 (0.71)	1151 (0.71)	0.943	366 (0.71)	304 (0.59)	0.016
Prior renal disease, N (%)	Present	13,548 (26.32)	23,390 (14.42)	< 0.001	13,534 (26.31)	13,500 (26.25)	0.810
Cancer, N (%)	Present	2995 (5.82)	16,511 (10.18)	< 0.001	2993 (5.82)	3489 (6.78)	< 0.001
Acquired Immunodeficiency Syndrome, N (%)	Present	22 (0.04)	1448 (0.89)	< 0.001	22 (0.04)	26 (0.05)	0.564
Undergone any surgery, N (%)	Present	957 (1.86)	3991 (2.46)	< 0.001	954 (1.85)	1074 (2.09)	0.007
Bronchial fibroscopy, N (%)	Yes	232 (0.45)	1700 (1.05)	< 0.001	232 (0.45)	234 (0.45)	0.926
Chest computed tomography, N (%)	Yes	1894 (3.68)	9025 (5.57)	< 0.001	1891 (3.68)	2256 (4.39)	< 0.001
Dialysis, N (%)	Yes	381 (0.74)	1261 (0.78)	0.395	380 (0.74)	383 (0.74)	0.913
Oxygen prior to admission, N (%)	Present	3987 (7.74)	7946 (4.90)	< 0.001	3983 (7.74)	3434 (6.68)	< 0.001
Non-invasive lung ventilation, N (%)	Yes	1571 (3.05)	4121 (2.54)	< 0.001	1568 (3.05)	1377 (2.68)	< 0.001
Invasive lung ventilation, N (%)	Yes	755 (1.47)	3501 (2.16)	< 0.001	754 (1.47)	565 (1.10)	< 0.001
Days of hospital stay, median (IQR)		8 (7)	7 (7)	0.135	8 (7)	7 (7)	0.352
Mortality, N (%)	Yes	8089 (15.71)	17,843 (11.00)	< 0.001	8083 (15.72)	7990 (15.53)	0.425

After PSM, men with atrial fibrillation prior to hospital admission had more often suffered from heart failure (*p* < 0.001), cerebrovascular disease (*p* < 0.001), and more frequently used oxygen at home (*p* = 0.025), but less often had type 2 diabetes mellitus (*p* = 0.003) (Table 3). Despite they more commonly received both non-invasive and invasive lung ventilation (both *p* < 0.001), their IHM did not differ from IHM in men with no atrial fibrillation (14.90% vs. 14.99%; *p* = 0.631).

**Table 3:** Clinical characteristics, and in-hospital outcomes of men hospitalized with community-acquired pneumonia in Spain from 2016 to 2019 according to atrial fibrillation prior to hospital admission, before and after propensity score matching.

		Before propensity score matching			After propensity score matching		
		Atrial fibrillation prior to hospital admission			Atrial fibrillation prior to hospital admission		
		Yes	No	*p* value	Yes	No	*p* value
Age, mean (SD)		80.76 (9.14)	71.15 (15.84)	< 0.001	80.76 (9.14)	80.84 (9.28)	0.354
Age category, N (%)	18–54	800 (1.11)	36,893 (15.76)	< 0.001	797 (1.11)	791 (1.10)	0.978
	55–69	7510 (10.44)	54,158 (23.13)		7494 (10.43)	7487 (10.42)	
	70–84	35,720 (49.64)	93,047 (39.74)		35,666 (49.63)	35,746 (49.74)	
	≥ 85	27,929 (38.81)	50,062 (21.38)		27,907 (38.83)	27,840 (38.74)	
Charlson comorbidity index, mean (SD)		2.65 (1.94)	2.25 (2.16)	< 0.001	2.65 (1.94)	2.73 (2.05)	0.114
Prior myocardial infarction, N (%)	Present	5900 (8.20)	13,333 (5.69)	< 0.001	5890 (8.20)	5870 (8.17)	0.847
Prior congestive heart failure, N (%)	Present	29,469 (40.95)	33,108 (14.14)	< 0.001	29,414 (40.93)	28,513 (39.68)	< 0.001
Prior peripheral vascular disease, N (%)	Present	6847 (9.52)	15,553 (6.64)	< 0.001	6836 (9.51)	6,682 (9.30)	0.164
Prior cerebrovascular disease, N (%)	Present	6267 (8.71)	14,496 (6.19)	< 0.001	6258 (8.71)	5441 (7.57)	< 0.001
Dementia, N (%)	Present	5592 (7.77)	16,883 (7.21)	< 0.001	5587 (7.77)	5714 (7.95)	0.213
Prior Chronic obstructive pulmonary disease, N (%)	Present	31,008 (43.09)	85,003 (36.30)	< 0.001	30,964 (43.09)	31,189 (43.40)	0.231
Type 2 diabetes mellitus, N (%)	Present	24,064 (33.44)	62,698 (26.78)	< 0.001	24,021 (33.43)	24,562 (34.18)	0.003
Prior rheumatoid disease, N (%)	Present	1615 (2.24)	3977 (1.70)	< 0.001	1614 (2.25)	1449 (2.02)	0.003
Prior peptic ulcer, N (%)	Present	484 (0.67)	1605 (0.69)	0.715	483 (0.67)	529 (0.74)	0.147
Prior liver disease, N (%)	Present	4100 (5.70)	17,924 (7.65)	< 0.001	4093 (5.70)	3646 (5.07)	< 0.001
Prior hemiplegia or paraplegia, N (%)	Present	509 (0.71)	2248 (0.96)	< 0.001	509 (0.71)	489 (0.68)	0.525
Prior renal disease, N (%)	Present	20,131 (27.98)	38,842 (16.59)	< 0.001	20,106 (27.98)	20,440 (28.44)	0.050
Cancer, N (%)	Present	9205 (12.79)	40,453 (17.28)	< 0.001	9190 (12.79)	10,241 (14.25)	< 0.001
Acquired Immunodeficiency Syndrome, N (%)	Present	101 (0.14)	3585 (1.53)	< 0.001	100 (0.14)	166 (0.23)	< 0.001
Undergone any surgery, N (%)	Present	1948 (2.71)	7381 (3.15)	< 0.001	1936 (2.69)	2010 (2.80)	0.232
Bronchial fibroscopy, N (%)	Yes	694 (0.96)	3296 (1.41)	< 0.001	693 (0.96)	574 (0.80)	0.001
Chest computed tomography, N (%)	Yes	3965 (5.51)	16,221 (6.93)	< 0.001	3953 (5.50)	4222 (5.87)	0.002
Dialysis, N (%)	Yes	1014 (1.41)	2943 (1.26)	0.002	1009 (1.40)	986 (1.37)	0.604
Oxygen prior to admission, N (%)	Present	6186 (8.60)	15,066 (6.43)	< 0.001	6177 (8.60)	5941 (8.27)	0.025
Non-invasive lung ventilation, N (%)	Yes	2369 (3.29)	6603 (2.82)	< 0.001	2359 (3.28)	1988 (2.77)	< 0.001
Invasive lung ventilation, N (%)	Yes	1821 (2.53)	6873 (2.94)	< 0.001	1817 (2.53)	1492 (2.08)	< 0.001
Days of hospital stay, median (IQR)		7 (7)	7 (7)	0.365	7 (7)	7 (7)	0.563
Mortality, N (%)	Yes	10,725 (14.90)	27,352 (11.68)	< 0.001	10,705 (14.90)	10,770 (14.99)	0.631

### Clinical characteristics and in-hospital outcomes for women and men by atrial fibrillation diagnosed during hospital admission status after propensity score matching

After PSM, women with atrial fibrillation diagnosed during hospital admission had more often had a myocardial infarction (*p* < 0.001) (Table 4). During hospital admission, they more commonly underwent surgery, dialysis, and received both non-invasive and invasive lung ventilation (all p values < 0.001). Their IHM was higher than IHM in women with no atrial fibrillation during hospital admission (24.37% vs. 13.36%; *p* < 0.001).

**Table 4:** Clinical characteristics, and in-hospital outcomes of women hospitalized with community-acquired pneumonia in Spain from 2016 to 2019 according to atrial fibrillation diagnosed during hospital admission, before and after propensity score matching.

		Before propensity score matching			After propensity score matching		
		Atrial fibrillation diagnosed during hospital admission			Atrial fibrillation diagnosed during hospital admission		
		Yes	No	*p* value	Yes	No	*p* value
Age, mean (SD)		79.63 (11.56)	75.80 (16.27)	< 0.001	79.50 (11.64)	79.50 (11.62)	0.989
Age category, N (%)	18–54	35 (2.92)	25,497 (12.00)	< 0.001	35 (3.04)	35 (3.04)	0.999
	55–69	193 (16.08)	34,272 (16.13)		187 (16.22)	187 (16.22)	
	70–84	488 (40.67)	72,929 (34.33)		466 (40.42)	467 (40.50)	
	≥ 85	484 (40.33)	79,733 (37.53)		465 (40.33)	464 (40.24)	
Charlson comorbidity index, mean (SD)		2.18 (1.76)	1.79 (1.78)	< 0.001	2.17 (1.76)	2.06 (1.65)	0.703
Prior myocardial infarction, N (%)	Present	72 (6.00)	5967 (2.81)	< 0.001	69 (5.98)	36 (3.12)	0.001
Prior congestive heart failure, N (%)	Present	513 (42.75)	53,928 (25.39)	< 0.001	485 (42.06)	485 (42.06)	0.998
Prior peripheral vascular disease, N (%)	Present	44 (3.67)	5116 (2.41)	0.005	41 (3.56)	30 (2.60)	0.185
Prior cerebrovascular disease, N (%)	Present	91 (7.58)	11,968 (5.63)	0.004	84 (7.29)	70 (6.07)	0.243
Dementia, N (%)	Present	111 (9.25)	24,077 (11.33)	0.023	105 (9.11)	104 (9.02)	0.942
Prior Chronic obstructive pulmonary disease, N (%)	Present	268 (22.33)	47,599 (22.41)	0.951	258 (22.38)	258 (22.38)	1.000
Type 2 diabetes mellitus, N (%)	Present	331 (27,58)	53,110 (25.00)	0.039	316 (27.41)	316 (27.41)	1.000
Prior rheumatoid disease, N (%)	Present	51 (4.25)	7918 (3.73)	0.341	48 (4.16)	44 (3.82)	0.670
Prior peptic ulcer, N (%)	Present	9 (0.75)	850 (0.40)	0.056	9 (0.78)	4 (0.35)	0.164
Prior liver disease, N (%)	Present	67 (5.58)	9079 (4.27)	0.025	63 (5.46)	44 (3.82)	0.060
Prior hemiplegia or paraplegia, N (%)	Present	12 (1.00)	1506 (0.71)	0.231	11 (0.95)	14 (1.21)	0.546
Prior renal disease, N (%)	Present	267 (22.25)	36,671 (17.26)	< 0.001	253 (21.94)	254 (22.03)	0.960
Cancer, N (%)	Present	121 (10.08)	19,385 (9.13)	0.251	119 (10.32)	98 (8.50)	0.134
Acquired Immunodeficiency Syndrome, N (%)	Present	2 (0.17)	1468 (0.69)	0.028	2 (0.17)	0 (0.00)	0.157
Undergone any surgery, N (%)	Present	114 (9.50)	4834 (2.28)	< 0.001	111 (9.63)	34 (2.95)	< 0.001
Bronchial fibroscopy, N (%)	Yes	19 (1.58)	1913 (0.90)	0.013	19 (1.65)	10 (0.87)	0.093
Chest computed tomography, N (%)	Yes	87 (7.25)	10,832 (5.10)	0.001	84 (7.29)	67 (5.81)	0.152
Dialysis, N (%)	Yes	50 (4.17)	1592 (0.75)	< 0.001	49 (4.25)	15 (1.30)	< 0.001
Oxygen prior to admission, N (%)	Present	57 (4.75)	11,876 (5.59)	0.206	53 (4.60)	61 (5,.29)	0.442
Non-invasive lung ventilation, N (%)	Yes	118 (9.83)	5574 (2.62)	< 0.001	115 (9.97)	19 (1.65)	< 0.001
Invasive lung ventilation, N (%)	Yes	158 (13.17)	4098 (1.93)	< 0.001	157 (13.62)	26 (2.25)	< 0.001
Days of hospital stay, median (IQR)		12 (12)	7 (7)	< 0.001	12 (12)	8 (7)	< 0.001
Mortality, N (%)	Yes	287 (23.92)	25,645 (12.07)	< 0.001	281 (24.37)	154 (13.36)	< 0.001

After PSM, men with atrial fibrillation diagnosed during hospital admission had more often had a myocardial infarction (*p* < 0.001) (Table 5). During hospital admission, they more commonly underwent surgery, bronchial fibroscopy, dialysis, and received both non-invasive and invasive lung ventilation (all p values < 0.001). Their IHM was higher than IHM in men with no atrial fibrillation during hospital admission (23.94% vs. 14.04%; *p* < 0.001).

**Table 5:** Clinical characteristics, and in-hospital outcomes of men hospitalized with community-acquired pneumonia in Spain from 2016 to 2019 according to atrial fibrillation diagnosed during hospital admission, before and after propensity score matching.

		Before propensity score matching			After propensity score matching		
		Atrial fibrillation diagnosed during hospital admission			Atrial fibrillation diagnosed during hospital admission		
		Yes	No	*p* value	Yes	No	*p* value
Age, mean (SD)		74.66 (11.36)	73.40 (15.13)	< 0.001	74.53 (11.40)	74.56 (11.39)	0.946
Age category, N (%)	18–54	97 (4.96)	37,596 (12.36)	< 0.001	94 (5.06)	93 (5.00)	0.999
	55–69	490 (25.08)	61,178 (20.11)		474 (25.50)	473 (25.44)	
	70–84	953 (48.77)	127,814 (42.02)		899 (48.36)	900 (48.41)	
	≥ 85	414 (21.19)	77,577 (25.50)		392 (21.09)	393 (21.14)	
Charlson comorbidity index, mean (SD)		2.78 (2.17)	2.34 (2.12)	< 0.001	2.76 (2.18)	2.72 (2.13)	0.351
Prior myocardial infarction, N (%)	Present	191 (9.77)	19,042 (6.26)	< 0.001	181 (9.74)	108 (5.81)	< 0.001
Prior congestive heart failure, N (%)	Present	643 (32.91)	61,934 (20.36)	< 0.001	588 (31.63)	588 (31.63)	0.996
Prior peripheral vascular disease, N (%)	Present	186 (9.52)	22,214 (7.30)	< 0.001	175 (9.41)	177 (9.52)	0.911
Prior cerebrovascular disease, N (%)	Present	137 (7.01)	20,626 (6.78)	0.687	128 (6.89)	141 (7.58)	0.411
Dementia, N (%)	Present	75 (3.84)	22,400 (7.36)	< 0.001	70 (3.77)	70 (3.77)	1,000
Prior Chronic obstructive pulmonary disease, N (%)	Present	781 (39.97)	115,230 (37.88)	0.058	737 (39.64)	736 (39.59)	0.973
Type 2 diabetes mellitus, N (%)	Present	598 (30.60)	86,164 (28.33)	0.026	555 (29.85)	553 (29.75)	0.943
Prior rheumatoid disease, N (%)	Present	37 (1.89)	5555 (1.83)	0.825	36 (1.94)	27 (1.45)	0.253
Prior peptic ulcer, N (%)	Present	22 (1.13)	2067 (0.68)	0.017	21 (1.13)	22 (1.18)	0.878
Prior liver disease, N (%)	Present	158 (8.09)	21,866 (7.19)	0.126	151 (8.12)	117 (6.29)	0.031
Prior hemiplegia or paraplegia, N (%)	Present	17 (0.87)	27,40 (0.90)	0.886	17 (0.91)	18 (0.97)	0.865
Prior renal disease, N (%)	Present	444 (22.72)	58,529 (19.24)	< 0.001	419 (22.54)	420 (22.59)	0.969
Cancer, N (%)	Present	413 (21.14)	49,245 (16.19)	< 0.001	398 (21.41)	383 (20.60)	0.546
Acquired Immunodeficiency Syndrome, N (%)	Present	10 (0.51)	3676 (1.21)	0.005	9 (0.48)	7 (0.38)	0.616
Undergone any surgery, N (%)	Present	256 (13.10)	9073 (2.98)	< 0.001	244 (13.13)	89 (4.79)	< 0.001
Bronchial fibroscopy, N (%)	Yes	54 (2.76)	3936 (1.29)	< 0.001	53 (2.85)	13 (0.70)	< 0.001
Chest computed tomography, N (%)	Yes	207 (10.59)	19,979 (6.57)	< 0.001	195 (10.49)	159 (8.55)	0.044
Dialysis, N (%)	Yes	134 (6.86)	3823 (1.26)	< 0.001	129 (6.94)	37 (1.99)	< 0.001
Oxygen prior to admission, N (%)	Present	126 (6.45)	21,126 (6.95)	0.389	117 (6.29)	131 (7.05)	0.357
Non-invasive lung ventilation, N (%)	Yes	254 (13.00)	8718 (2.87)	< 0.001	244 (13.13)	44 (2.37)	< 0.001
Invasive lung ventilation, N (%)	Yes	376 (19.24)	8318 (2.73)	< 0.001	372 (20.01)	79 (4.25)	< 0.001
Days of hospital stay, median (IQR)		13 (13)	7 (7)	< 0.001	13 (13)	7 (7)	< 0.001
Mortality, N (%)	Yes	465 (23.80)	37,612 (12.37)	< 0.001	445 (23.94)	261 (14.04)	< 0.001

### Multivariable analysis of factors associated with in-hospital mortality during admission for CAP among patients with atrial fibrillation prior to hospital admission

The risk of dying during hospital admission for CAP among patients with atrial fibrillation prior to hospital admission increased with age and most comorbidities, but chronic obstructive pulmonary disease (OR 0.77; 95% CI 0.74–0.80) and type 2 diabetes mellitus (OR 0.90; 95% CI 0.87–0.93) were associated with a lower IHM (Table 6). Whilst undergoing chest computed tomography was associated with a lower IHM (OR 0.70; 95% CI 0.65–0.77), dialysis (OR 2.17; 95% CI 1.91–2.47), non-invasive lung ventilation (OR 2.65; 95% CI 2.45–2.85) and invasive lung ventilation (OR 6.75; 95% CI 6.16–7.40) were associated with a higher IHM (ORs and 95% CIs are for the overall population). Male sex was associated with a higher IHM (OR 1.06; 95% CI 1.02–1.10).

**Table 6:** Multivariable analysis of factors associated with in-hospital mortality during admission for community-acquired pneumonia among patients with atrial fibrillation prior to hospital admission, according to sex.

Variable	Male	Female	Both
	**Odds ratio (95% confidence interval)**		
Age 18–54 years	1	1	1
Age 55–69 years	1.43 (1.08–1.88)	1.93 (1.08–3.46)	1.50 (1.17–1.92)
Age 70–84 years	2.24 (1.71–2.93)	3.67 (2.08–6.50)	2.46 (1.93–3.13)
Age ≥ 85	4.08 (3.12–5.34)	7.08 (4.00–12.53)	4.59 (3.60–5.85)
Prior myocardial infarction		1.15 (1.02–1.30)	1.07 (1.01–1.14)
Prior congestive heart failure	1.20 (1.15–1.25)	1.11 (1.06–1.17)	1.16 (1.12–1.20)
Prior cerebrovascular disease	1.43 (1.33–1.53)	1.56 (1.44–1.69)	1.49 (1.41–1.57)
Dementia	1.89 (1.77–2.20)	1.79 (1.68–1.91)	1.84 (1.76–1.93)
Prior chronic obstructive pulmonary disease	0.76 (0.73–0.80)	0.78 (0.73–0.83)	0.77 (0.74–0.80)
Type 2 diabetes mellitus	0.89 (0.85–0.94)	0.90 (0.85–0.95)	0.90 (0.87–0.93)
Prior rheumatoid disease		1.15 (1.01–1.31)	
Prior liver disease	1.18 (1.08–1.30)		1.16 (1.08–1.26)
Prior hemiplegia or paraplegia	1.73 (1.40–2.14)	2.06 (1.63–2.61)	1.88 (1.60–2.20)
Prior renal disease	1.18 (1.12–1.23)	1.19 (1.13–1.26)	1.18 (1.14–1.23)
Cancer	2.22 (2.09–2.34)	2.01 (1.83–2.21)	2.16 (2.06–2.27)
AIDS		3.26 (1.15–9.25)	
Undergone any surgery	1.15 (1.02–1.30)		1.15 (1.04–1.27)
Chest computed tomography	0.73 (0.66–0.82)	0.64 (0.55–0.75)	0.70 (0.65–0.77)
Dialysis	2.04 (1.75–2.37)	2.57 (2.01–3.27)	2.17 (1.91–2.47)
Oxygen prior to admission	1.26 (1.16–1.36)	1.26 (1.16–1.38)	1.26 (1.19–1.34)
Non-invasive lung ventilation	2.62 (2.38–2.89)	2.69 (2.39–3.02)	2.65 (2.45–2.85)
Invasive lung ventilation	6.80 (6.09–7.58)	6.68 (5.66–7.89)	6.75 (6.16–7.40)
Male sex	Not applicable	Not applicable	1.06 (1.02–1.10)

Odds ratios indicate those variables significantly associated with in-hospital mortality. Blank spaces denote variables excluded in the final model.

### Multivariable analysis of factors associated with in-hospital mortality during admission for CAP among patients with atrial fibrillation diagnosed during hospital admission

The risk of dying during hospital admission for CAP among patients with atrial fibrillation diagnosed prior to hospital admission increased with age and most comorbidities (Table 7). Whilst undergoing chest computed tomography was associated with a lower IHM (OR 0.67; 95% CI 0.48–0.93), dialysis (OR 3.01; 95% CI 2.14–4.25), non-invasive lung ventilation (OR 1.79; 95% CI 1.39–2.31) and invasive lung ventilation (OR 3.21; 95% CI 2.50–4.11) were associated with a higher IHM (ORs and 95% CIs are for the overall population). Male sex was not associated with a higher IHM (OR 0.93; 95% CI 0.77–1.13).

**Table 7:** Multivariable analysis of factors associated with in-hospital mortality during admission for community-acquired pneumonia among patients with atrial fibrillation diagnosed during hospital admission, according to sex.

Variable	Male	Female	Both
	**Odds ratio (95% confidence interval)**		
Age 18–54 years	1	1	1
Age 55–69 years	1.98 (1.06–3.69)	0.73 (0.29–1.89)	1.46 (0.88–2.43)
Age 70–84 years	2.74 (1.47–5.09)	1.65 (0.67–4.08)	2.30 (1.39–3.79)
Age ≥ 85	2.94 (1.50–5.75)	2.45 (0.96–6.23)	2.93 (1.73–4,97)
Prior myocardial infarction	1.62 (1.14–2.30)		1.49 (1.11–2.00)
Prior congestive heart failure	1.38 (1.08–1.75)		1.26 (1.05–1.52)
Prior cerebrovascular disease		1.79 (1.10–2.92)	
Dementia	2.51 (1.49–4.24)		1.58 (1.12–2.23)
Prior rheumatoid disease	2.60 (1.26–5.37)		
Cancer	2.02 (1.54–2.65)	1.66 (1.04–2.64)	1.91 (1.52–2.40)
Chest computed tomography	0.62 (0.41–0.93)		0.67 (0.48–0.93)
Dialysis	3.29 (2.18–4.97)	2.53 (1.30–4.94)	3.01 (2.14–4.25)
Non-invasive lung ventilation	1.66 (1.21–2.28)	1.95 (1.25–3.05)	1.79 (1.39–2.31)
Invasive lung ventilation	3.28 (2.44–4.41)	3.67 (2.27–5.95)	3.21 (2.50–4.11)
Male sex	Not applicable	Not applicable	0.93 (0.77–1.13)

Odds ratios indicate those variables significantly associated with in-hospital mortality. Blank spaces denote variables excluded in the final model.

## Discussion

Here we found that almost one quarter of the people older than 17 years admitted to the hospital for CAP had atrial fibrillation prior to hospital admission, but less than one percent had new onset atrial fibrillation during hospital admission. Versus no atrial fibrillation, IHM was significantly higher in people with atrial fibrillation prior to hospital admission, and length of hospital stay and IHM were higher in people with atrial fibrillation diagnosed during hospital admission. After PSM, women and men with atrial fibrillation prior to hospital admission received both non-invasive and invasive lung ventilation more often than women and men with no atrial fibrillation, but their IHM did not differ (around 15% for each group). Women and men with atrial fibrillation diagnosed during hospital admission more commonly underwent surgery, dialysis, and received both non-invasive and invasive lung ventilation, yet their IHM was much higher than IHM in women and men with no atrial fibrillation. The risk of dying during hospital admission for CAP among patients with both atrial fibrillation prior to hospital admission or diagnosed during hospital admission increased with age, comorbidities, and interventional procedures. Age, comorbidities and invasive lung ventilation were the strongest predictive factors of IHM, in line with previous reports^14^.

Male sex was associated with a higher IHM in patients with atrial fibrillation prior to hospital admission, but sex did not modify IHM in patients with atrial fibrillation diagnosed during hospital admission.

Almost one quarter of the population admitted for CAP had prevalent atrial fibrillation in our study. This figure is higher than previous reports^15,16^ and we believe that it basically depends on the age of the population included, the quality of the coding process and the qualification of “present on admission” even for patients with clinical history of paroxysmal atrial fibrillation. Contrarily, we found a remarkably low rate of coding for atrial fibrillation diagnosed during hospital admission (less than 1%), and again it probably reflects our definition for the variable: atrial fibrillation onset during hospital admission and “not present on admission”. This definition essentially rules out patients who presented at the ED with sinus rhythm but had previously had atrial fibrillation in its paroxysmal form but were coded as “present on admission”. Not surprisingly, these are the patients who present episodes of atrial fibrillation during admission for CAP more frequently.^7^ In previously published research, this number also depended on the baseline characteristics of the population studied, since in patients admitted to the intensive care unit for CAP this number raised significantly^17^. Several mechanisms for new onset of atrial fibrillation have been proposed, like hypoxia, heart scarring or rises in cytosolic calcium, which affects endothelial cadherin junctions thus inducing apoptosis that leads to cardiac injury and arrhythmia^18^.

Both meteorological conditions and the high altitude may have some influence on the occurrence of paroxysms of atrial fibrillation^19,20^. We could not adjust for specific meteorological conditions because in order to guarantee the confidentiality and privacy of the information the Spanish Register of Specialized Care-Basic Minimum Database (RAE-CMBD) does not include geographic information about the hospital or the place of residence of the patients. However, this circumstance would not probably affect atrial fibrillation developed during hospitalization. Only extreme altitude has been reported to exert some effect on the onset of arrhythmias^19^; besides, long-term high-altitude exposure does not seem to increase the incidence of atrial fibrillation, at least that associated with organic heart diseases^21^. Anyway, it is estimated that less than 50,000 people in Spain live over 1500 m above the sea level, that is, less than 0.1% of the population^22^.

In our study, patients with either atrial fibrillation prior to hospital admission or diagnosed during hospital admission received both non-invasive and invasive lung ventilation more often than patients with no atrial fibrillation. In our investigation after the multivariate analysis, invasive mechanical ventilation turns to be the strongest predictive factor of mortality with an adjusted OR of 6.80 (95% CI 6.09–7.58) for males and 6.68 (95% CI 5.66–7.89) for females with atrial fibrillation prior to hospital. The results of Espinoza et al. who analyzed 802 patients admitted to ICUs with a diagnosis of CAP showed an adjusted OR for mechanical ventilation of 5.07 (95% CI 5.54–7.27)^23^.

Given the important association of invasive ventilation with IHM is surprising that in prevalent AF patients, after PSM no IHM differences were found for males of females even if these patients needed more ventilation. In our opinion, probably, the increased IHM risk associated with more frequent invasive mechanical ventilation among women with prevalent atrial fibrillation is counterbalanced by a higher prevalence of dementia, cancer and any surgery among women without prevalent atrial fibrillation. The increased IHM risk associated with more frequent invasive mechanical ventilation among men with prevalent atrial fibrillation is probably counterbalanced by a higher prevalence of prior renal disease, cancer and AIDS among men without prevalent atrial fibrillation.

The Preventive Medicine Department at each hospital is responsible for ensuring the adherence to the strict protocols established for the reutilization of sanitary material and to prevent nosocomial infections, according to European and Spanish Legislation^24^. Atrial fibrillation may contribute to hemodynamic instability during an acute infection probably signaling adrenergic overstimulation^25^. It seems that a worse clinical situation during admission prompted the indication of a higher number of procedures in a population with an a priori higher probability of death during the hospital stay.

Versus no atrial fibrillation, we detected no differences in IHM in people with atrial fibrillation prior to hospital admission, but a higher IHM in patients with new onset atrial fibrillation during hospital admission for CAP. The association between new onset atrial fibrillation and mortality in severely ill patients has been described by many authors^26,27^. Whether new onset of atrial fibrillation is a marker of higher clinical severity, of distinct pathophysiologic mechanisms, or deleterious by itself or by the therapeutic measures that its incidence calls for cannot be clarified with the design of our study. IHM was as high as ≈ 24% in people who developed atrial fibrillation during hospital admission. We had no access to information on end of life decisions, but surely a policy of palliative care was followed in many cases. This highlights the need to develop skills beyond technical knowledge to talk with the patients and their relatives, understand their psychological needs and prepare them for the possibility of a clinical course that does not fulfill the expectations.

In this study we could see that male sex was associated with a higher IHM in patients with atrial fibrillation prior to hospital admission, but not in patients with atrial fibrillation diagnosed during hospital admission. We have previously reported higher IHM in males for CAP in the Spanish population^13^, but we do not figure out why we are only seeing this gender gap in the case of atrial fibrillation prior to hospital admission. We were able to adjust for baseline clinical congestive heart failure, but we could not account for chronic left ventricular ejection fraction. We might hypothesize that lower baseline values of this parameter in men could have a negative impact on mortality even in patients with no previous clinical decompensation of heart failure to explain this heterogeneous effect associated with gender. Notwithstanding this argument, the incidence of mortality due to CAP in heart failure patients seems to be higher with preserved left ventricular ejection fraction^28^. Another explanation is that a potentially higher IHM in male is counterbalanced by the higher rate of use of both non-invasive and invasive mechanical ventilation in men in the case of atrial fibrillation diagnosed during hospital admission that we are reporting here, whereas rates of use of mechanical ventilation among men and women with atrial fibrillation prior to hospital admission were quite similar in our population. When non-invasive mechanical ventilation is used and works since the outset, it is accepted that it confers a survival advantage in CAP^29^.

An unexpected result of our investigation was the protective odds of diabetes and COPD in the IHM after CAP among patients with atrial fibrillation prior to hospital admission.

However, diabetes has been associated with a lower IHM in previous studies of CAP conducted in our country^30^. Suggested explanations for this association are that patients with diabetes are hospitalized with a less severe disease or that the presence of obesity, a condition very frequent in people with diabetes, could explain this lower mortality. The existence of a ‘obesity survival paradox’ for pneumonia has been reported in meta-analysis and observational reports^31,32^.

Also the lower mortality of patients with COPD after CAP has been described in previous investigations^30,33^. The selection bias, previously commented for patients with diabetes, could result in those patients with COPD being more likely to be hospitalized with less severe pneumonia. Other possible reasons are that, given the overlap in symptoms/clinical findings between COPD exacerbations and pneumonia, exacerbations could be mistakenly coded as CAP. This misclassification has been suggested by other authors when ICD10 codes are used^34^. Finally, this could also be due to a protective anti-inflammatory effect of inhaled corticosteroids and different immune responses secondary to an altered microbiome in COPD subjects^35–37^.

For both, diabetes and COPD patients, another possible explanation would be an earlier diagnosis or treatment initiation in these patients. However, future studies, with more detailed clinical information, are required to clarify these associations.

Our investigation has strengths and limitations that must be considered. The external validity is the most relevant strength as we cover almost all hospital discharges for an entire country with a constant methodology over a four-year period^38,39^.

Regarding limitations, as in most hospital based administrative discharge databases, we lack information on laboratory results, radiological images, treatments with antibiotic, anticoagulants or anti-platelet therapy prior or within the hospital, lifestyles (smoking, obesity, physical exercise) and severity scales for pneumonia^38,39^. Secondly, the presence of residual confounding cannot be ruled out even if the PSM may have helped to reduce it. Third, we don’t have data on the after hospital discharge mortality. It is possible that if a patient is transferred from one hospital to another with the same diagnosis would be counted twice, however due to the severity of CAP we think this is extremely infrequent and would not affect our results. Finally, to our knowledge, no external validation has been performed to assess the validity of the identified predictive factors and diagnosis codes using an external dataset.

In conclusion, our study shows an association between atrial fibrillation diagnosed during hospital admission and IHM in people admitted for CAP, but not for atrial fibrillation prior to hospital admission. Whether new onset of atrial fibrillation is a marker of higher clinical severity or deleterious by itself needs to be elucidated.

## Methods

### Study population

We included in the study every episode of hospital admission for CAP in Spanish people older than 17 years. We used data for the period January 1st, 2016-December 31st, 2019, from the RAE-CMBD. Additional details on the RAE-CMBD can be found online^40^. The International Classification of Disease, Tenth Revision (ICD-10) guided the codification of discharge diagnoses and therapeutic procedures. The codes used to identify patients hospitalized with CAP are defined in Supplementary Table S1. The study population was stratified according to sex, in a similar fashion to previous research^38^.

### Study variables

We sought atrial fibrillation codes (ICD10-codes 148.xx) among people admitted for CAP. The “Present on Admission (POA)” indicator enabled us to discriminate between patients who had been diagnosed with atrial fibrillation before the index hospitalization and patients who developed atrial fibrillation during the hospitalization period.

For those patients admitted more than once over the study period only the first episode was analyzed and the rest of hospital admissions discarded.

The main outcomes study variable was the IHM.

As proposed by Sundararajan et al. the Charlson Comorbidity Index (CCI) was used to assess the comorbidites^41^. Other covariates included diagnostic and therapeutic procedures like use of oxygen prior to hospitalization, any surgical procedure during the hospital admission, bronchial fibroscopy, dialysis or computerized axial tomography. (Supplementary Table S1).

### Propensity score matching method

We used a PSM method to reduce potential residual confounding implicit to research work out of randomized clinical trials^42^. We matched each woman who had a code for atrial fibrillation prior to hospital admission with another woman of the same age and baseline clinical conditions with no atrial fibrillation, and we proceeded in a similar way for atrial fibrillation diagnosed during hospital admission. We adhered to the same criteria for the matching process among men. Multivariable logistic regression was used to estimate the PS for each patient that was then matched to the patient with the closest PS value in the corresponding non-atrial fibrillation subpopulation The matching variables were age and the comorbid conditions present at admission.

### Statistical analysis

Absolute frequencies and proportions are reported for categorical variables and means or medians, with standard deviations (SDs) and interquartile ranges (IQRs) respectively, for continuous variables.

To assess significant differences between study subgroups we used chi-square, t test or the Mann–Whitney test before PSM and McNemar’s test and a paired t test after PSM^42^.

Variables independently associated with IHM were identified constructing multivariable logistic regression models replicating the steps proposed by Hosmer et al.^43^. We constructed models separately for men and women Finally, we analyzed the effect of sex in two models: 1. Patients with atrial fibrillation prior to hospital admission; and 2. Patients with atrial fibrillation diagnosed during hospital admission. The results were expressed as odds ratios (ORs) with their 95% confidence intervals (CIs).

Stata version 14 (Stata, College Station, Texas, USA) was the software used for all statistical analysis.

### Ethics

The RAE-CMBD is owned by the Spanish Ministry of Health and can be accessed upon request^44^. This registry is anonymized and under public access, which means that according to Spanish legislation, approval by an ethics committee can be waived. As the RAE-CMBD is an administrative database the information of all the patients hospitalized is mandatory by law so there is no need to ask patients for informed consent, since it is assumed that if the patient agrees to be hospitalized, they are implicitly giving their consent for their data to be included in anonymized administrative databases.

## Supplementary Information

Supplementary Information.

## Data Availability

"No additional data available". According to the contract signed with the Spanish Ministry of Health and Social Services, which provided access to the databases from the Spanish National Hospital Discharge Database (SNHDD), we cannot share the databases with any other investigator, and we must delete the databases once the investigation has concluded. Consequently, we cannot upload the databases to any public repository. However, any investigator can apply for access to the databases by filling out the questionnaire available at: http://www.msssi.gob.es/estadEstudios/estadisticas/estadisticas/estMinisterio/SolicitudCMBDdocs/Formulario_Peticion_Datos_CMBD.pdf. All other relevant data are included in the paper.
