# Supplementary material for: Analysis of the association between atrial fibrillation with in-hospital mortality in people admitted for community-acquired pneumonia through an observational, nation-wide, sex-stratified study
Source: Sci Rep. 2022 Aug 24;12:14404. doi: 10.1038/s41598-022-18810-8 (PMC9402531; doi:10.1038/s41598-022-18810-8)
Supplement: Supplementary file 1 — Supplementary Information. [file 41598_2022_18810_MOESM1_ESM.docx]

**Supplemental Table S1**. International Classification of Diseases-10 (ICD-10) codes for diagnosis and therapeutic procedures used in this investigation.

|  | **ICD-10 codes** |
| --- | --- |
| Community-acquired pneumonia* | J12 to J18 as primary diagnosis with a POA indicator of “Y”.  J12 to J18 in any of the secondary diagnosis fields (2-20) and with a POA indicator of “Y”. |
| Atrial fibrillation present at admission | 148.xx as primary diagnosis with a POA indicator of “Y”.  148.xx in any of the secondary diagnosis fields (2-20) and with a POA indicator of “Y”. |
| Atrial fibrillation diagnosed during the hospital admission | 148.xx in any of the secondary diagnosis fields (2-20) and with a POA indicator of “N”. |
| Bronchial fibroscopy | 0BJ08ZZ |
| Computerized axial tomography of thorax | BW24 |
| Dialysis | 5A1D00Z, 5A1D60Z,3E1M39Z |
| Oxygen prior to hospitalization | Z99.81 |
| Non-invasive mechanical ventilation | 5A09357, 5A09457, 5A09557 |
| Invasive mechanical ventilation | 5A1945Z, 5A1955Z, 5A1935Z |
